# Predicting kidney injury after cardiac surgery with cardio-pulmonary bypass using machine learning

**DOI:** 10.3389/fdgth.2026.1695494

**Published:** 2026-02-26

**Authors:** Janis Fliegenschmidt, Gunung Sasono, Patricia Cabanillas Silva, Laurent Meesseman, Mohamed Rezk, Michael Dahlweid, Astrid Bergmann, Nikolai Hulde, Vera von Dossow

**Affiliations:** 1 Institute for Anaesthesiology and Pain Therapy, Herz- und Diabeteszentrum NRW, Ruhr-Universität Bochum, Bad Oeynhausen, Germany; 2 Institute for Anaesthesiology and Pain Therapy, Herz- und Diabeteszentrum NRW, Med. Fakultät OWL (Universität Bielefeld), Bad Oeynhausen, Germany; 3 Dedalus HealthCare, Antwerp, Belgium; 4 Dedalus SpA, Milan, Italy; 5 Sino-European Joint Lab for Health Information Processing and Applications, Jiaxing University, Jiaxing, China

**Keywords:** acute kidney injury, artificial intelligence, cardiac surgery, machine learning, risk prediction

## Abstract

**Introduction:**

Acute kidney injury after cardiac surgery (CSA-AKI) is a common complication after cardiac surgery and an independent predictor of morbidity and mortality. Since evidence-based care is based on risk mitigation and implementation of supportive measures, early risk stratification and consequent adjustment of treatment strategies are elemental. Artificial intelligence screening can aid in pre- and perioperative risk stratification.

**Methods:**

This is a secondary analysis of 130 prospectively recruited patients from one center of a multicenter observational trial that investigated the implementation of a bundle of supportive measures to prevent AKI in patients undergoing cardiac surgery with cardiopulmonary bypass. Machine learning (ML) enabled artificial intelligence (AI) was used to retrospectively analyze patients' electronic health record (EHR) data and generate an AKI risk estimate. The aim of this study was to investigate the feasibility of AI-based risk scores to predict AKI within 72 hours postoperatively and the development of acute kidney disease (AKD) at day 30 after surgery.

**Results:**

Of 130 patients, 33.1% developed CSA-AKI. Of 119 with 30-day follow-up data, 18.5% developed AKD. Day-of-surgery AI risk-scoring was evaluated with an AUROC of 0.79 for occurrence of CSA-AKI, postoperative risk predictions were evaluated with an AUROC of 0.83 for AKD at 30 days postoperatively. ANOVA testing revealed that patients who developed CSA-AKI or AKD had significantly higher predicted risk scores than those who did not, with large effect sizes. Predicted risk also increased significantly over the perioperative period in patients with adverse outcomes.

**Conclusions:**

Risk stratification with an ML-based AI approach based solely on EHR data provides a low-effort and high-yield screening method for identifying patients at risk of developing CSA-AKI and AKD. These findings indicate that an EHR (Electronic Health Record)-only model, trained on routine hospital data, provides clinically actionable discrimination in a real-world cardiac surgery cohort, supporting its use for early screening and targeted mitigation.

## 1 Introduction

Postoperative acute kidney injury (AKI) is a frequent and serious complication of surgical interventions in general (1), and in cardiac surgery in particular (cardiac surgery associated AKI, CSA-AKI), where 20%–30% of patients are affected by postoperative AKI (2). The occurrence of AKI after surgery is associated with several short- and long-term complications, prompting Lei et al. to term it a “sentinel complication” due to its association with other postoperative complications, prolonged hospitalization, higher mortality, and development of chronic kidney disease (CKD) (1). The increased risk of developing CKD after CSA-AKI has a significant impact on the patient's health and quality of life, while also placing a considerable burden on the healthcare system (3). This is especially a concern as the demographic shift in developing countries leads to an increased age average in the patient collective undergoing cardiac surgery. These patients, due to frailty and comorbidity, are increasingly vulnerable to postoperative complications from cardiac surgery, including CSA-AKI (3).

AKI is a syndromic condition that, in the context of cardiac surgery, is most likely induced by a variety of factors. Several mechanisms of injury can plausibly occur from both the surgery and the pathology that warranted intervention in the first place. These include ischemia, hypoperfusion-reperfusion injury, hemolysis, embolization, and systemic inflammation (4). Mechanisms as well as biomarkers are the subject of ongoing research. However, the definition of AKI has been largely standardized to the “Kidney Disease: Improving Global Outcomes” (KDIGO) criteria in 2012 (5), which has helped researchers agree on a common presentation and improved awareness to the high incidence of AKI after surgery by incorporating urine output as a diagnostic criterion (6).

After diagnosis of AKI, evidence-based treatment is limited to supportive care. Many interventions aim at modifying risk factors, which could be implemented as part of preoperative preparation and perioperative care. Early risk stratification and strategic adjustment are essential in helping to prevent the development of manifest CSA-AKI and reduce associated morbidity and mortality (3, 7, 8).

Machine Learning (ML) approaches to event prediction can be applied to the prediction of complications of medical care. Event prediction is one of the core applications for artificial intelligence (AI) in perioperative care (9). Establishing proactive risk mitigation protocols instead of reactive complication control is a unique opportunity with AI based risk stratification (10). Implementing AI screening based on electronic health record (EHR) data could enable low-effort, highly available risk screening with real-time response to changes in patient data. In contrast to established risk scores like the Cleveland Clinic score (11), the presented AI scoring tool does not require manual evaluation and considers a multitude of factors, enabling continuous risk monitoring that can serve as preoperative risk stratification, but also alert to an increasing risk in the postoperative period. Additionally, it stratifies risk for AKI of any severity.

After promising results with postoperative delirium screening (12, 13), this study evaluates an implemented, CE-certified, EHR-only, hospital integrated AI screening system based on ML techniques towards the risk stratification of CSA-AKI and AKD (defined as reduced kidney function after 72 h or 30 days, respectively) on prospectively collected data from patients undergoing cardiac surgery with cardiopulmonary bypass (CPB). The model development itself is not part of this work, but has been extensively described in earlier publications (14–17).

## 2 Methods

This study was conducted at the Heart- and Diabetes Centre NRW (HDZ NRW), a high-volume cardiac surgery center in Germany. Ethical approval for this study was provided by the Ethics Committee of the Medical Faculty, reference number 2023-1045 on 28th February 2023, Ruhr-University Bochum, Division OWL (PO Box 10 03 61, 32503 Bad Oeynhausen).

### 2.1 Study participants

Patients that were included in the “Implementation of the KDIGO Guidelines for the Prevention of AKI in Patients after Cardiac Surgery”-study (8) at HDZ NRW were eligible for this study (*n* = 143). These were patients between 18 and 90 years of age with no preexisting AKI, who were scheduled for cardiac surgery with CPB. Exclusion criteria included pre-existing AKI and CKD with eGFR 20 < mL/min/1.73 m^2^ or dependence on dialysis (refer to Supplementary Table S2 for the full list). Additionally, only patients with a complete set of prediction data were included in this analysis (*n* = 130, Supplementary Figure S1). The last exclusion criterion is the only additional criterion in this secondary analysis. It effected the removal of four datasets.

### 2.2 Patient data acquisition

Patients were screened for eligibility on admission and asked for written informed consent prior to their surgery. Data were collected prospectively from surgery until 72 h postoperatively, as well as at the 30-day follow-up. Clinical data on demographics and perioperative care were recorded from chart reviews. The primary endpoint of the index study was the proportion of patients treated with the KDIGO Guideline Bundle, secondary endpoints included the incidence and severity of AKI at 72 h postoperatively and after 30 days, the need for renal replacement therapy, the length of patients' intensive care unit (ICU) stay, and the overall duration of their hospitalization. AKI was diagnosed and staged by an experienced anesthesiologist using the urine output and serum creatinine changes according to the KDIGO criteria. Additional information was programmatically extracted from the digital anesthesia records, specifically: Left ventricular ejection fraction (LVEF), body mass index (BMI), preoperative serum creatinine (SCr), and preoperative hemoglobin concentration (Hb).

These data were gathered only for the evaluation in this study; the AI-model could only access the EHR and was not specifically supplied with any manually curated information.

### 2.3 Artificial intelligence predictions

Artificial intelligence predictions were generated using the clinalytix Medical AI clinical risk prediction model^1^ for AKI. The ML-based prediction model was trained on ten years of historical EHR data from HDZ (*n* = 91,290, using a case-control ratio of 1:5; see also Supplementary Table S1), previously published in (14). To avoid any kind of data leakage, previous information about the study cohort was completely removed from model development. No model parameters, hyperparameters, or decision thresholds were tuned or fitted on this cohort. The labels were assigned based on the KDIGO creatinine change criteria. Urine output was not available for the labelling of the training cases and therefore not used. All predictions were generated from the data gathered in the hospital's EHR system. These were comprised of structured data, such as laboratory results, vital signs, and historical records, along with unstructured data, such as clinical notes. The latter were automatically analyzed by the AI software using Natural Language Processing (NLP) to identify the presence of clinical entities. Anesthesia records and intensive care charts were not included. A CatBoost (Categorical Boosting tree classifier) model architecture was used to train a binary classifier for the prediction of AKI (18). The same trained model was used to predict CSA-AKI and acute kidney disease (AKD).

The prediction software generated a chronological stream of patient data during their stay so to have a fully versioned copy of their patient chart. This representation allowed for the retrospective calculation of risk predictions without the introduction of information leakage. Predictions were generated after every change in a patient's record, meaning that any individual patient had multiple updates to their risk score during the day. The analysis in this study is based on the highest prediction score of the respective day, or series of days.

The generated risk score is the probability that the patient will develop the event based on the given data. Warning levels of “increased” and “high” risk (0.5 and 0.75, respectively) were arbitrarily chosen. As the risk probabilities in this study were generated retrospectively, these warnings were neither available to the clinicians treating the patients nor to the clinician evaluating the patients for AKI for the original study.

All preprocessing and feature handling (encoding, missing data handling, and regularization) were defined during initial model development and documented in prior work (14–17): Before data preparation, we applied the following inclusion criteria: (1) patients should be 18 years or older and must have a birth year available; (2) medical cases should contain information about the gender, the department, and the reason for admission; and (3) stay in the hospital should be for a maximum of 90 days. Next, input features were generated using a common data preparation pipeline, which used all the available data generated during the patient's stay. The features came from structured data, such as laboratory results, vital signs, and historical records, along with unstructured data, such as clinical notes. The model accesses the same feature domains for all patients (see also Supplementary Table S3). In practice, not all measurements exist for every individual; the model is designed to handle such sparsity internally. No additional feature selection or tuning was performed on the 130-patient validation cohort. A summary Shapley analysis of the 20 most important features is depicted in Supplementary Figure S4.

### 2.4 Statistical analysis

Statistical analysis was performed using the tidyverse 1.3.2 (19), ggplot2 3.3.6 (20), and effsize 0.8.1 (21) packages in R 4.3.1 (22). Summary statistics were calculated using base R and tidyverse capabilities. Tests were considered significant at the 95% confidence level. Group differences were analyzed using the Mann–Whitney-*U* test (MWU) or Chi-Squared test (X^2^) where appropriate, and Bonferroni correction was applied. Boxplot graphics were generated with ggplot2 to demonstrate the distribution of the AI risk probabilities. Differences in predicted risks between groups were calculated using a Two-way, repeated measures Analysis of Variance (ANOVA) test with base R. The effect size of the inter-group difference was calculated using Cohen's *d* from the effsize package. Plotting of receiver operating characteristic (ROC) curves and area under the receiver operating characteristic curve (AUROC) calculations were performed with Python 3.11.4 and the scikit-learn 1.3.2 (23) package to demonstrate discrimination performance independent of whether predictions are well-calibrated.

## 3 Results

### 3.1 Patient characteristics of the study cohort

The 130 patients included in the study were at a mean age of 64.1 ± 11.8 [Mean ± standard deviation (SD)] years. 33.1% of patients developed CSA-AKI within 72 h after surgery and 18.5% of patients developed AKD within 30 days after surgery. 58.3% of patients with AKD within 30 days also had manifest CSA-AKI within 72 h. Table 1 summarizes baseline patient characteristics stratified by occurrence of kidney injury at either (or both) timepoints. Notably, patients who developed kidney injury were significantly older (68.9 ± 9.7 vs. 60.8 ± 12.1 years, *p* < 0.01, MWU), had higher baseline serum creatinine (1.07 ± 0.33 vs. 0.88 ± 0.23 mg/dL, *p* < 0.01, MWU), and lower LVEF (51.1 ± 11.0 vs. 58.1 ± 6.7%, *p* < 0.01, MWU). Other variables, including sex distribution, Haemoglobin levels and BMI, did not exhibit statistically significant differences between groups. Surgeries were categorized into single valve procedures, multi-valve procedures, aortic surgery procedures [including aortic valve (AV) plus ascending aortic aneurysm (AAA) repair], combination procedures [valve plus coronary artery bypass graft (CABG), myectomy, lung resection, or AAA repair (only in combination with other valves than AV)], and re-operation surgeries. Thirty-day follow-up data was missing for eleven patients, three of which had been diagnosed with CSA-AKI.

**Table 1:** Overview of patient characteristics (prior to surgery) by incidence of CSA-AKI within 72 h or AKD within 30 days after cardiac surgery.

Variable	CSA-AKI/AKD	No kidney injury	Total
# of patients	53 (40.8%)	77 (59.2%)	130
Age (Mean ± SD) [years]	68.9 ± 9.7	60.8 ± 12.1	64.1 ± 11.8
sex (% Female)	18/53 (34.0%)	27/77 (35.1%)	45/130 (34.6%)
Surgical intervention			
Single Valve	22 (34.9%)	42 (65.1%)	64
Multi-Valve	4 (44.4%)	5 (55.6%)	9
Combination	11 (39.3%)	16 (60.7%)	27
Aortic	8 (53.3%)	7 (46.7%)	15
Re-Operation	8 (53.3%)	7 (46.7%)	15
BMI (Mean ± SD) [kg/m^2^]	28.3 ± 5.7	27.2 ± 4.4	27.7 ± 5.0
LVEF (Mean ± SD) [%]	51.1 ± 11.0	58.1 ± 6.7	55.1 ± 9.4
SCr (Mean ± SD) [mg/dl]	1.07 ± 0.33	0.88 ± 0.23	0.95 ± 0.29
Hb (Mean ± SD) [mg/dl]	13.2 ± 2.1	13.7 ± 1.5	13.5 ± 1.8

CSA-AKI, cardiac surgery associated acute kidney injury; AKD, acute kidney disease; SD, standard deviation; BMI, body mass index; LVEF, left ventricular ejection fraction; SCR, serum creatinine; HB, hemoglobin.

### 3.2 Risk prediction results

The predicted probability by the AI was generally higher for patients who suffered a decline in their kidney function as defined by the KDIGO criteria. The evaluation was performed for two prediction scenarios: (1) The ability of the highest risk score on the day of the operation to predict AKI within 72 h (CSA-AKI), and (2) the ability of the highest risk score within 72 h postoperatively to predict reduced kidney function within 30 days after surgery (AKD) (see Table 2).

**Table 2:** Summary of the key characteristics and results for the investigated prediction targets.

Outcome	CSA-AKI	AKD
Prediction Target	Reduced kidney function within 72 h of surgery	Reduced kidney function 30 d after surgery
Prediction Timeframe	On the day of the surgery	Within 72 h after surgery
Group Difference (ANOVA)	*F*_1,627_ = 81.981, *p* < 0.001	*F*_1,627_ = 81.349, *p* < 0.001
Effect Size of the group difference (Cohen's *d*)	0.98, 95% CI = [0.81; 1.16]	1.29, 95% CI = (1.08; 1.50)
Effect of Evaluation Time (ANOVA)	*F*_4,627_ = 7.321, *p* < 0.001	*F*_4,627_ = 7.477, *p* < 0.001
Interaction between Group Difference and Evaluation Time (ANOVA)	*F*_4,627_ = 0.051, *p* = 0.995	*F*_4,627_ = 0.561, *p* = 0.691
AUROC	*0.79, 95% CI = (0.70; 0.86)*	*0.83, 95% CI = (0.73; 0.92)*

CSA-AKI, cardiac surgery associated acute kidney injury; AKD, acute kidney disease, ANOVA, analysis of variance; AUROC, area under the receiving operator characteristic.

#### 3.2.1 Predicting CSA-AKI

**Figure 1:**
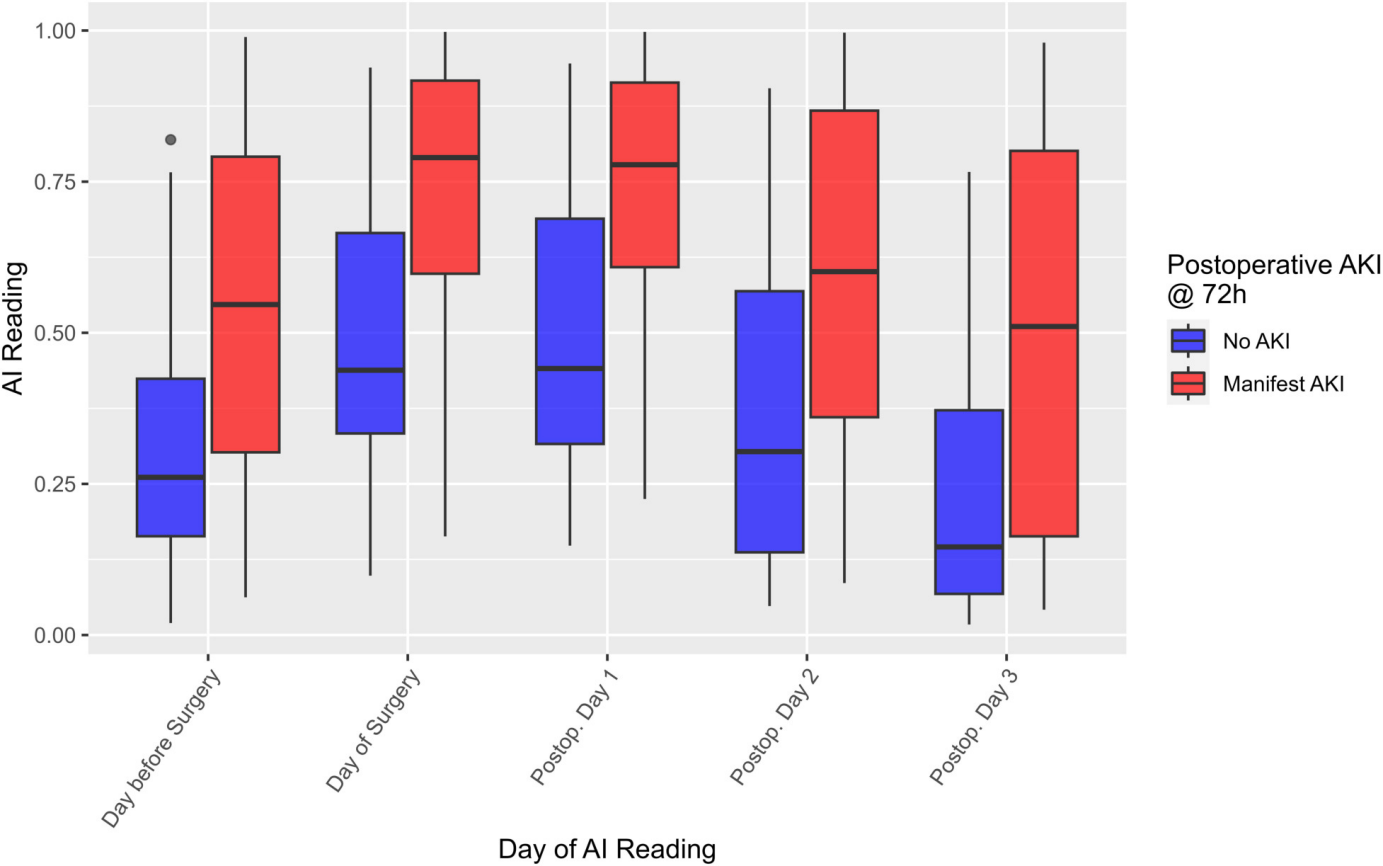
AI risk prediction scores during the perioperative period, distinguished by occurrence of CSA-AKI within 72 h of surgery. The CSA-AKI group (red) demonstrates a clear shift compared to the non-AKI group (blue), consistent with a large effect size (Cohen's *d* = 0.98). AI, artificial intelligence; CSA-AKI, cardiac surgery associated acute kidney injury.

**Figure 2:**
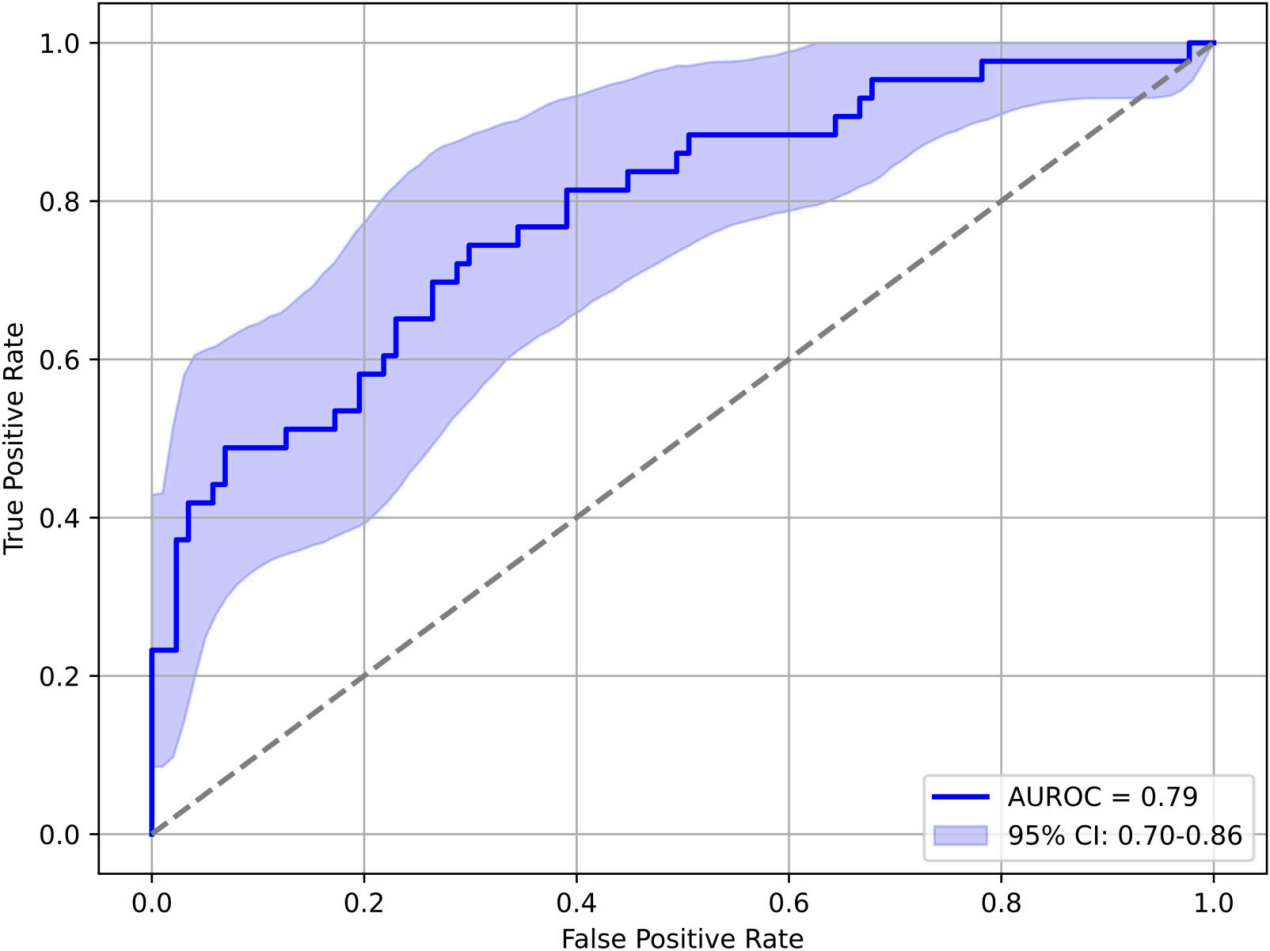
AUROC analysis of predicting CSA-AKI within 72 h after surgery from the highest AI risk Reading on the day of the operation. AUC, area under the curve; AUROC, area under the receiver operating characteristic curve; CSA-AKI, cardiac surgery associated acute kidney injury.

CSA-AKI within 72 h was predicted based on the highest AI risk prediction on the day of the operation. Figure 1 shows the dispersion of the risk score in the perioperative timeframe distinguished by patients who developed CSA-AKI within 72 h of surgery vs. patients who did not. Figure 2 shows the corresponding AUROC curve analysis. The Two-way, repeated measures ANOVA revealed statistically significant differences between the CSA-AKI groups (*F*_1,627_ = 81.981, *p* < 0.001) with a large effect size [Cohen's *d* = 0.98, 95% CI = (0.81; 1.16)], and a statistically significant effect of evaluation time (*F*_4,627_ = 7.321, *p* < 0.001), and no effect of an interaction between the two (*F*_4,627_ = 0.051, *p* = 0.995). A breakdown of warning levels (akin to the warning thresholds explained above) and KDIGO stages is shown in Table 3.

**Table 3:** AI warning level compared to KDIGO level of CSA-AKI. The warning level from the highest AI risk Reading from the day of the surgery is put against the highest KDIGO score within 72 h after the surgery, respectively.

Outcome	AI risk ≤ 0.5	0.5 < AI risk ≤ 0.75	AI risk > 0.75
No CSA-AKI	46	25	16
KDIGO Grade 1	7	9	9
KDIGO Grade 2	0	3	7
KDIGO Grade 3	0	1	7

AI, artificial intelligence; CSA-AKI, cardiac surgery associated acute kidney injury.

#### 3.2.2 Predicting AKD within 30 days postoperatively

**Figure 3:**
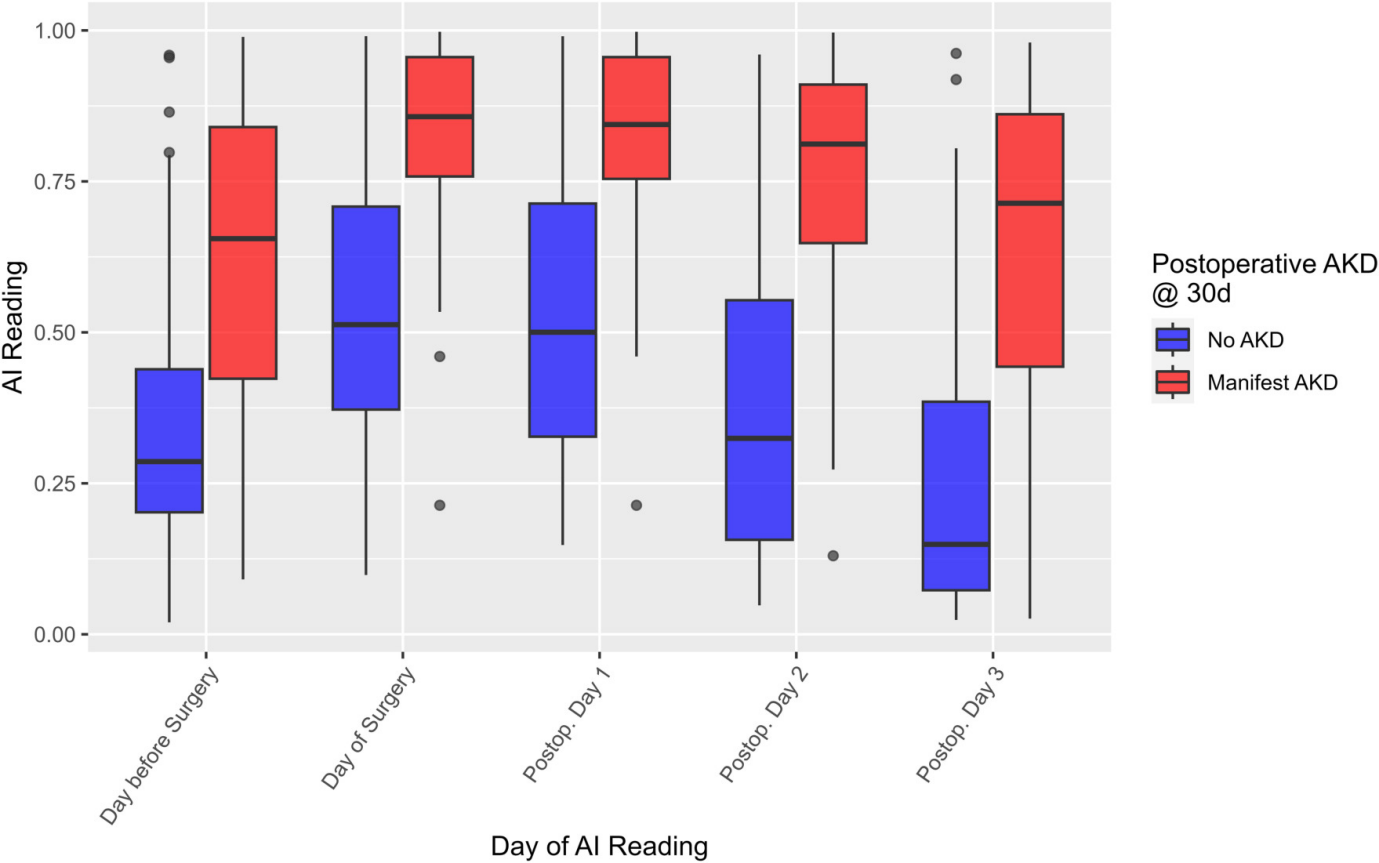
AI risk prediction scores during the perioperative period, distinguished by occurrence of AKD within 30 days of surgery. The AKD group (red) again demonstrates a clear shift compared to the non-AKD group (blue), consistent with a large effect size (Cohen's *d* = 0.98). AI, artificial intelligence; AKD, acute kidney disease.

**Figure 4:**
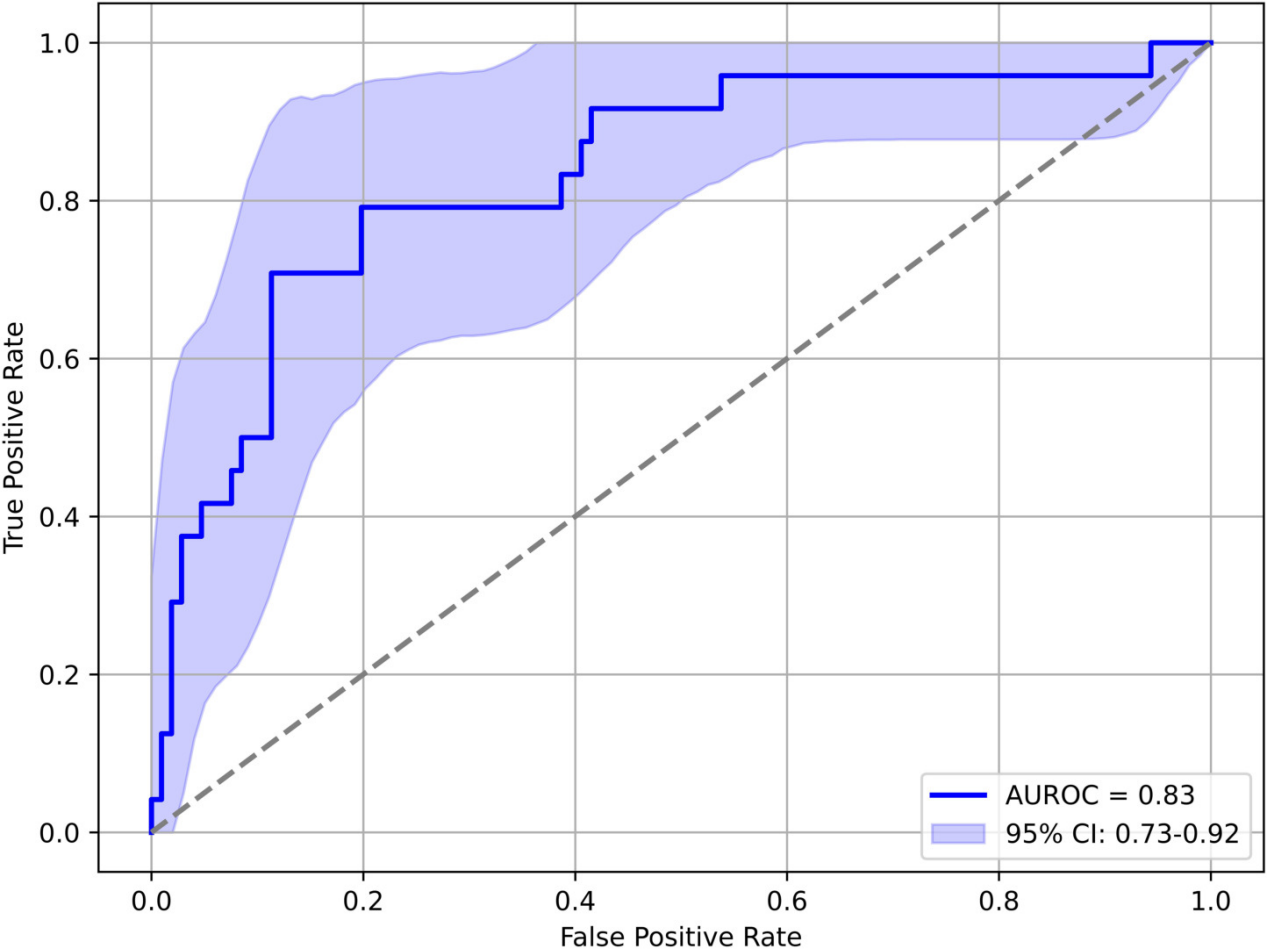
AUROC curve analysis of predicting AKD within 30 days after surgery from the highest AI risk Reading within three days postoperatively. AUC, area under the curve; AUROC, area under the receiver operating characteristic curve; AKD, acute kidney disease.

Reduced kidney function within 30 days was predicted based on the highest AI-predicted risk within three days after the operation. Figure 3 shows the risk probabilities in the same timeframe as Figure 1 but distinguished by occurrence of AKD within 30 days of their intervention. Figure 4 shows an AUROC curve analysis of predicting AKD within 30 days from the highest AI risk score within three days postoperatively. Similarly to the CSA-AKI case, for AKD the Two-way, repeated measures ANOVA revealed statistically significant differences between the groups (*F*_1,627_ = 81.349, *p* < 0.001) with a large effect size [Cohen's *d* = 1.29, 95% CI = (1.08; 1.50)], and a statistically significant effect of evaluation time (*F*_4,627_ = 7.477, *p* < 0.001), and no effect of an interaction between the two (*F*_4,627_ = 0.561, *p* = 0.691). Table 4 shows the respective warning level vs. KDIGO stage breakdown. Thirty-day follow-up data was not available for eleven cases, which were excluded from this analysis.

**Table 4:** AI warning level compared to KDIGO level of AKD within 30 days after cardiac surgery. The highest AI Reading within 3 days postoperatively is compared with the highest KDIGO score within 30 days after the surgery, respectively.

Outcome	AI risk ≤ 0.5	0.5 < AI risk ≤ 0.75	AI score > 0.75
No CSA-AKI	47	28	20
KDIGO Grade 1	2	3	13
KDIGO Grade 2	0	1	1
KDIGO Grade 3	0	0	4

AI, artificial intelligence; AKD, acute kidney disease.

For both prediction targets, the risk scores clearly differ between patients with compared to patients without manifest CSA-AKI or AKD. The results show that the predicted probabilities were higher for the AKI positive group compared with the negative group, and that they significantly changed over the perioperative time course, with the increased risk from surgery seemingly reflected in both groups.

## 4 Discussion

The usefulness of ML-based risk alert systems in general has been discussed many times (9, 10), also for other complications of cardiac surgery (12, 13, 24), and for AKI in general (25, 26). In this investigation, AI was used to predict AKI specifically after cardiac surgery on cardio-pulmonary bypass, an area with substantial clinical importance given the high morbidity associated with post-operative renal dysfunction (1). This study demonstrates that an ML-based AI model, trained on routinely collected EHR data, can effectively stratify risk for developing CSA-AKI following cardiac surgery with CPB. The strengths and limitations of the presented work are as follows.

### 4.1 High predictive performance and robustness

Although the AI model was not specifically trained for post-operative AKI, the generated predictions demonstrated robust discriminative ability for CSA-AKI within 72 h post-operation and AKD prediction within 30 days after surgery, using only perioperative data available in the hospital's digital health records. ANOVA testing revealed significant differences between case and control groups in both prediction scenarios, confirming the model's ability to meaningfully differentiate between patients who developed kidney injury post-operation and those who did not. The validity of the inter-group differences was reinforced as well by the large effect sizes (Cohen's *d* > 0.9). The increased risk predictions in the perioperative timeframe in both groups reflect the model's ability to capture the insult from surgery.

The higher AUROC for 30-day AKD prediction is particularly noteworthy, suggesting that the model captures longer-term risk factors with greater accuracy. This differential performance may reflect the model's ability to identify subtle patterns predictive of sustained kidney dysfunction that might not manifest immediately post-surgery but evolve over time. Additionally, the limited availability of data from the preoperative time frame impacts the analysis: Since patients were normally only admitted a day before their elective surgery, and to a highly specialized center where there were usually no earlier periods of admission to draw data from, historical data was scarce throughout the data set.

### 4.2 Rigorous and high-quality validation

Previous studies similarly found good predictability of CSA-AKI by means of machine learning enabled risk stratification (27, 28). However, the AI tool in this study not only performs well in comparison to earlier models. The described validation methodology is exceptional in that it used a high-quality validation cohort with a gold labelling standard. For training, this study used real-life data from 10 years that allowed us to have an extensive training set of more than 50,000 medical cases. It is worth noting that these cases were just routine EHR entries as well that were not recorded with the intention of performing register studies, let alone training machine learning algorithms. The AI model was tested against prospectively collected data, on which an experienced critical care physician diagnosed kidney injury by applying the KDIGO criteria. This ensured validation with high-quality, clinically meaningful data. Furthermore, most of the previously described models are abstract proof of concept studies, whereas the model in this study had already been trained and validated in the clinic's patient data (14, 15), has now been validated in a real-world scenario and is already integrated into an EHR solution.

### 4.3 Practicality and clinical utility

A key strength of the prediction model lies in its reliance solely on EHR data, allowing for effortless implementation and seamless integration into existing clinical workflows. Moreover, the early risk prediction capability of the AI model provides a clinical window for intervention before kidney injury becomes clinically evident. This timing advantage represents a shift from reactive to proactive, patient-individualized risk management.

### 4.4 Limitations and mitigation

However, several methodological limitations must be acknowledged. For the clinician-assigned labels, AKI was identified from ICU records 3 days postoperatively and usually at 30 days from follow-up visit documentation when available. This creates a potential “gray area” during the interim period and after 30 days post-operation as kidney injury would not be captured in the outcome labels. Additionally, apart from the variations in the selection of *baseline* creatinine measurements, there are differences in the KDIGO implementation between the clinician-assigned labels and the labelling approach used in the AI development, as the urine output was not available to the AI software and therefore not used. It was, however, used for labelling by a clinician when the patient had urine output monitored, i.e., during their ICU stay. Incorporating urine output into the diagnostic criteria represents an essential feature of the KDIGO criteria, yet the availability of urine output measurements outside the ICU is scarce in real-world data. However, to address this limitation, we ran additional analyses where we defined the AKI onset using in-house KDIGO-labelling algorithms that rely only on the creatinine levels (5, 16, 17). The analysis results and the conclusions were in line with previous findings (Supplementary Figures S2, S3).

Another limitation of this study is the rather small cohort. As this was a secondary analysis, the number of cases available for validation had already been fixed. Similarly, the unavailability of intraoperative and intensive care data blinds the AI to a critical phase in the patient's clinical journey. Intraoperative factors have been reported to be relevant for the risk of developing AKI postoperatively (29, 30). They were not directly available in either data set, but only recognized when they were separately documented in the EHR, e. g. in a discharge letter.

### 4.5 Summary

Our findings suggest that AI-based AKI prediction systems may offer substantial value as clinical decision support tools in cardiac surgery. Offering an early insight into the risk of kidney injury could serve as the basis for early risk stratification, facilitating strategic planning to reduce the likelihood of unfavorable outcomes. Integration into EHR systems is key to realizing the full potential of such solutions by requiring no effort on part of the user to trigger the risk stratification process. Seamless integration is an essential step toward routine use.

## 5 Conclusion and future directions

This study demonstrates the real-world value of an automated clinical decision support system for predicting cardiac surgery-associated AKI (CSA-AKI). The model presented in this study operated independently of manual data entry or intraoperative monitoring, highlighting its practicality in real-world settings. The investigated model is an already CE-certified software product that has been developed to be individually adapted to deploying hospitals (14, 15) and integrated into an existing hospital information system, meaning that the software is ready to be deployed into clinical settings.

Since the present model operates solely on pre- and early postoperative EHR data, an important avenue for future improvement lies in the integration of intraoperative parameters—such as blood pressure variability, cardiopulmonary bypass duration, vasopressor use, and fluid management. These data, routinely captured in anesthesia information management systems, are known contributors to renal perfusion dynamics and may carry strong predictive signals for CSA-AKI. Incorporating them into the model could further enhance predictive accuracy and temporal resolution. A future study aimed at integrating and validating intraoperative data streams may not only refine risk stratification but also enable real-time intraoperative alerts, opening a new frontier for proactive, perioperative kidney protection strategies.

While promising, these findings must be interpreted in the context of a single-center, retrospective validation. Prospective studies are needed to assess the clinical utility of AI-guided risk stratification, accompanied by risk-adjusted management protocols, to evaluate impact on patient outcomes. Nevertheless, this work provides a critical foundation for future deployment of AI tools that support proactive renal protection strategies in high-risk surgical populations.

## Data Availability

The data analyzed in this study is subject to the following licenses/restrictions: GDPR and institutional guidelines. Requests to access these datasets should be directed to Institute for Anaesthesiology and Pain Therapy, HDZ NRW, anaesthesiologie@hdz-nrw.de.

## Footnotes

^1^clinalytix Medical AI, Dedalus Healthcare GmbH, CE number: (01)4260693990149(8012)5.1.0(11)250616.
