# Supplementary material for: Predicting kidney injury after cardiac surgery with cardio-pulmonary bypass using machine learning
Source: Front Digit Health. 2026 Feb 26;8:1695494. doi: 10.3389/fdgth.2026.1695494 (PMC12979440; doi:10.3389/fdgth.2026.1695494)
Supplement: Supplementary file 1 [file Datasheet1.docx]

# Supplemental Material

| \| Number of Records (Number of Patients) \| 91,290 (54,688) \| \| --- \| --- \| \| Age, median \| 65.1 \| \| Female Sex, count (%) \| 19,610 (36%) \| \| Elective Admission, count (%) \| 81,020 (89%) \| \| Emergency Admission, count (%) \| 9,640 (10%) \| \| History of diagnosis \| 36,680 (40%) \| \| Cases \| 24,558  KDIGO Stage 1: 22,229  KDIGO Stage 2: 1372  KDIGO Stage 3: 957 \|   Table S1: Training data characteristics. 54,688 sets of patient data were used to train the ML model. The data set was 1:5 balanced between patients with and without the outcome to be predicted. [14] |
| --- | --- | --- | --- | --- | --- | --- | --- | --- | --- | --- | --- | --- | --- | --- |

| \| Inclusion criteria: \| Exclusion criteria: \| \| --- \| --- \| \| Patients scheduled for cardiac surgery with CPB \| Pre-existing AKI (KDIGO stage 1 and higher) \| \| Age between 18-90 years \| Patients with cardiac assist devices (ECMO, LVAD, RVAD, IABP) \| \| Written informed consent \| Pregnancy, breastfeeding \| \|  \| Known (Glomerulo-) Nephritis, interstitial nephritis or vasculitis \| \|  \| CKD with eGFR < 20 ml/min/1.73m2 \| \|  \| Dialysis dependent CKD \| \|  \| Prior kidney transplant within the last 12 months \| \|  \| Persons held in an institution by legal or official order \| \|  \| Persons with any kind of dependency on the investigator or employees by the investigator \|   Table S2: Inclusion and exclusion criteria of the index study. CPB: Cardiopulmonary bypass, AKI: Acute kidney injury, ECMO: Extra-corporal membrane oxygenation, LVAD: Left ventricular assist device, RVAD: Right ventricular assist device, IABP: Intra-aortic balloon pump, CKD: Chronic kidney disease. |
| --- | --- | --- | --- | --- | --- | --- | --- | --- | --- | --- | --- | --- | --- | --- | --- | --- | --- | --- | --- | --- |

| \| Feature group - Feature value \| \| # used \| \| --- \| --- \| --- \| \| **Gender** \| \|  \| \|  \| Male \| 2 \| \|  \| Female \| \| **Age group** \| \|  \| \|  \| 1 [18, 25] \| 9 \| \|  \| 2 [26, 35] \| \|  \| 3 [36, 45] \| \|  \| 4 [46, 55] \| \|  \| 5 [56, 65] \| \|  \| 6 [66, 75] \| \|  \| 7 [76, 85] \| \|  \| 8 [86, 95] \| \|  \| 9 ≥ 95 \| \| **ICD historical** \| \| 2759 \| \| **ICD from clinical text** \| \| 2584 \| \| **Medication** \| \| 4346 \| \| **Medication from clinical text** \| \| 5640 \| \| **Lab results** \| \|  \| \|  \| LL (very low) \| 15 \| \|  \| L (low) \| 99 \| \|  \| N (normal) \| 0 \| \|  \| H (high) \| 138 \| \|  \| HH (very high) \| 37 \| \| **Surgery duration** \| \|  \| \|  \| 5_30MIN \| 6 \| \|  \| 30_60MIN \| \|  \| 60_120MIN \| \|  \| 120_240MIN \| \|  \| 240_360MIN \| \|  \| 360_1440MIN \| \| **Surgery Asepsis level** \| \|  \| \|  \| 1_CLEAN \| 4 \| \|  \| 2_CLEAN_CONTAMINATED \| \|  \| 3_CONTAMINATED \| \|  \| 4_DIRTY_INFECTED \| \| **Surgery ICPM** \| \| 1012 \| \| Total \|  \| 16651 \| |
| --- | --- | --- | --- | --- | --- | --- | --- | --- | --- | --- | --- | --- | --- | --- | --- | --- | --- | --- | --- | --- | --- | --- | --- | --- | --- | --- | --- | --- | --- | --- | --- | --- | --- | --- | --- | --- | --- | --- | --- | --- | --- | --- | --- | --- | --- | --- | --- | --- | --- | --- | --- | --- | --- | --- | --- | --- | --- | --- | --- | --- | --- | --- | --- | --- | --- | --- | --- | --- | --- | --- | --- | --- | --- | --- | --- | --- | --- | --- | --- | --- | --- | --- | --- | --- | --- | --- | --- | --- | --- | --- | --- | --- | --- | --- | --- | --- | --- |
| Table S3: Breakdown of feature types in the EHR environment the model was trained and evaluated in at HDZ NRW. ICD: International Classification of Diseases, ICPM: International Classification of Procedures in Medicine. |

| 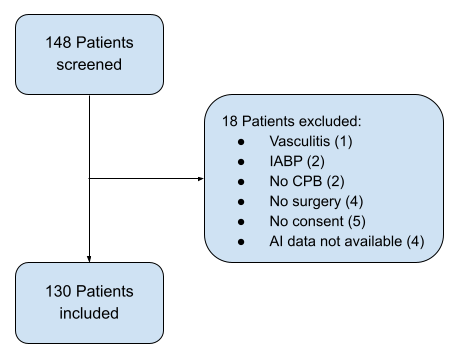 |
| --- |
| Figure S1: Patient inclusion pathway. IABP: Intra-aortic balloon pump, AI: Artificial intelligence. |

| 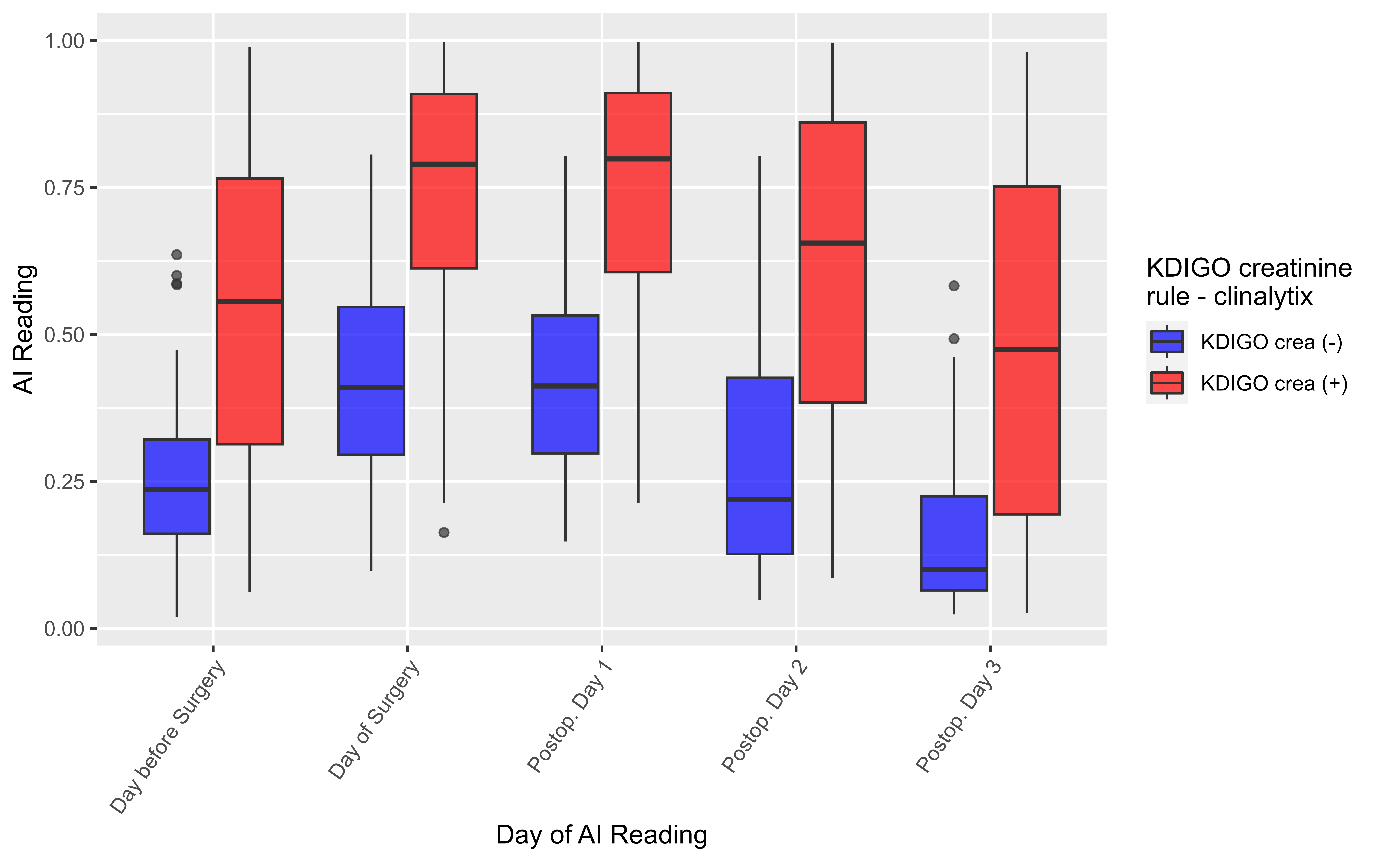 |
| --- |
| Figure S2: AI risk prediction scores during the perioperative period, distinguished by the automatically assigned label (as done with the retrospectively labelled training cases): The KDIGO creatinine rule becoming positive during the stay. The AKI group (red) demonstrates a clear shift compared to the non-AKI group (blue) here as well. The Two-way, repeated measures ANOVA revealed statistically significant differences between the groups (F_(1,627)_=98.888, *p* < 0.001) with a large effect size (Cohen’s *d* = 1.25, 95% CI = [1.08; 1.42]), and a statistically significant effect of evaluation time (F_(4, 627)_ = 8.568, *p* < 0.001), and no effect of an interaction between the two (F_(4, 627)_ = 0.072, *p* = 0.991). KDIGO: Kidney disease improving global outcome, AI: Artificial intelligence, AKI: Acute kidney injury. |

| 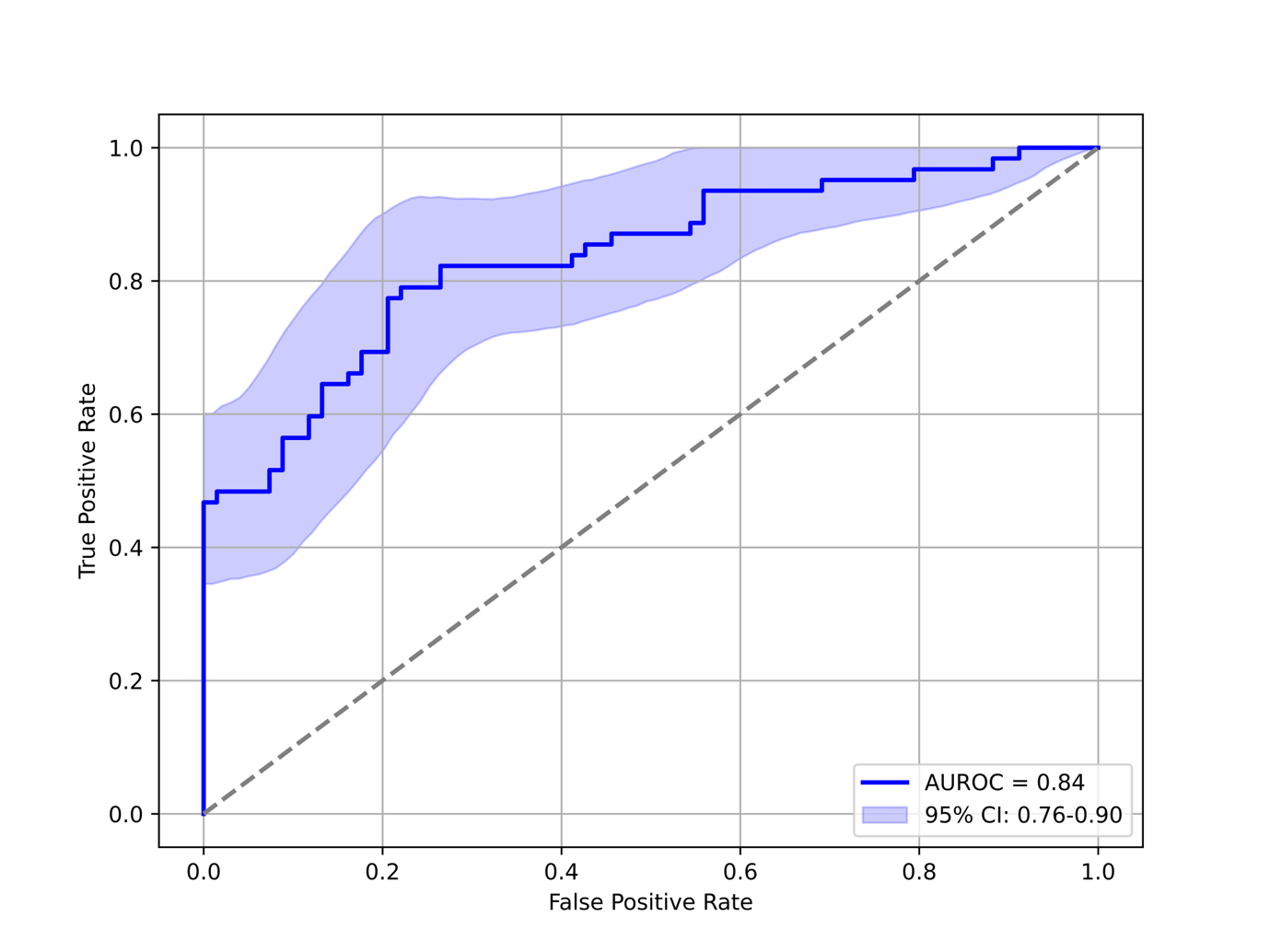 |
| --- |
| 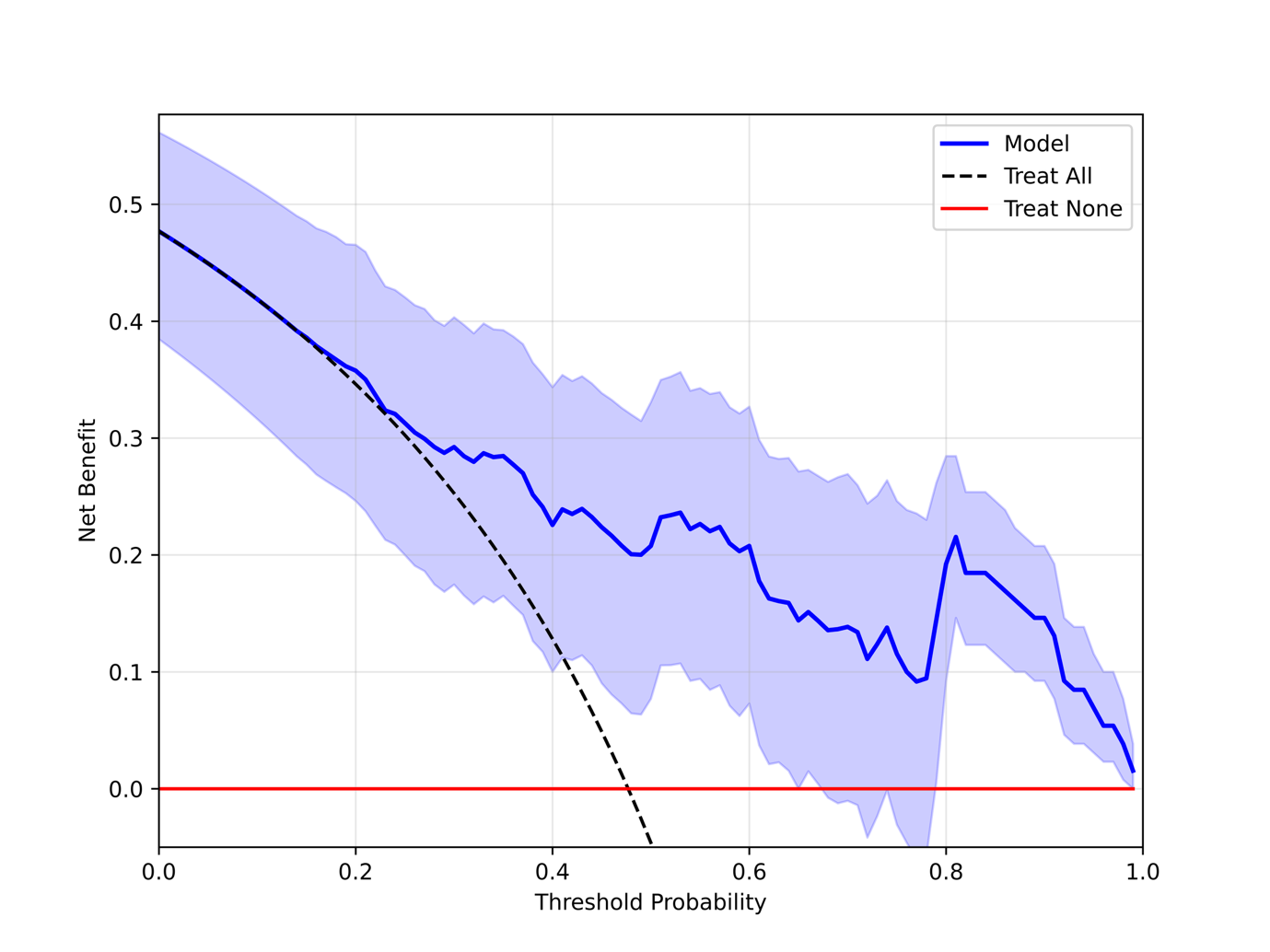 |
| Figure S3. *Top:* AUROC curve analysis of predicting AKI, as labelled by the creatinine rule of the KDIGO criteria, from the highest AI risk reading within three days postoperatively.  *Bottom:* DCA of predicting AKI, as labelled by the creatinine rule of the KDIGO criteria, from the highest AI risk reading within three days postoperatively.  KDIGO: Kidney disease improving global outcome, AUC: Area under the curve, AUROC: Area under the receiver operating characteristic curve, DCA: Decision curve analysis. |

| **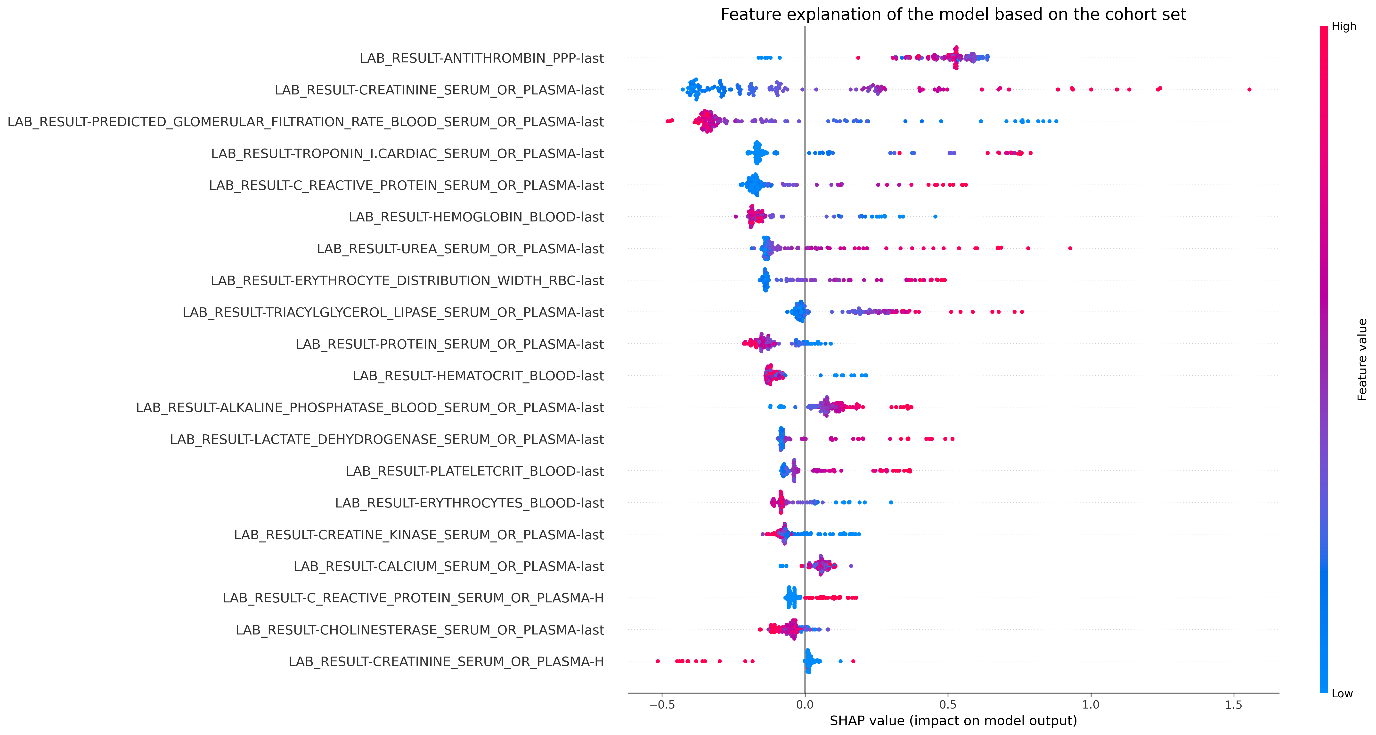** |
| --- |
| Figure S4: Shapley summary plot showing the distribution of feature’s impact on model output for the top 20 most important features. Each point represents an individual patient, with the color indicating the feature value (red for high, blue for low) and the position along the x-axis showing the SHAP value (impact on model prediction). |
